# Controversies in Screening and Diagnostic Criteria for Gestational Diabetes in Early and Late Pregnancy

**DOI:** 10.3389/fendo.2018.00696

**Published:** 2018-11-27

**Authors:** Evelyn A. Huhn, Simona W. Rossi, Irene Hoesli, Christian S. Göbl

**Affiliations:** ^1^Department of Obstetrics and Gynaecology, University Hospital Basel, Basel, Switzerland; ^2^Department of Biomedicine, University of Basel and University Hospital Basel, Basel, Switzerland; ^3^Division of Obstetrics and Feto-Maternal Medicine, Department of Obstetrics and Gynaecology, Medical University of Vienna, Vienna, Austria

**Keywords:** gestational diabetes (GDM), screening, diagnostic criteria, pregnancy, early biomarkers

## Abstract

This review serves to evaluate the screening and diagnostic strategies for gestational diabetes and overt diabetes in pregnancy. We focus on the different early screening and diagnostic approaches in first trimester including fasting plasma glucose, random plasma glucose, oral glucose tolerance test, hemoglobin A1c, risk prediction models and biomarkers. Early screening for gestational diabetes is currently not recommended since the potential benefits and harms of early detection and subsequent treatment need to be further evaluated in randomized controlled trials.

## Introduction

Gestational diabetes mellitus (GDM) is known to manifest in the second half of pregnancy in the setting of profound physiologic insulin resistance. Therefore, GDM is normally diagnosed after 24 weeks of gestation. The screening and diagnosis of GDM vary widely between medical specialties and among countries. Controversial areas surrounding screening for GDM include recommendations not to screen at all, a universal vs. a risk-based, selective approach, optimal timing of screening, the appropriate screening method [fasting plasma glucose (FPG), random plasma glucose (RPG), glucose challenge test (GCT)], or criteria for diagnosis (1 or 2 step, 75 vs. 100 g glucose load, whether 1 or 2 abnormal values are required for the diagnosis) and the appropriate cut-off values. Furthermore, there are debates concerning the relevance of treating additionally diagnosed, milder forms of GDM and about the cost effectiveness of different screening or diagnostic strategies. This article provides an update on screening and diagnostic strategies for GDM and overt diabetes. Furthermore, we will discuss the latest developments regarding early detection of GDM in the first trimester.

## The long-lasting way of developing screening and diagnostic criteria

After almost six decades of research and tremendous effort to reach a consensus a globally and uniformly accepted guideline regarding how and when to screen and diagnose GDM is still not available. The original criteria were established based on the 3-h 100 g OGTT by O‘Sullivan and Mahan in 1964 and predicted women who were most likely to develop type 2 diabetes mellitus (T2DM) later in life after pregnancy (1). But consecutive studies could show that even lesser degrees of hyperglycaemia were associated with an increased risk of adverse perinatal outcome, including large for gestational age fetuses, shoulder dystocia, neonatal hypoglycaemia, increased risk of cesarean section or hypertensive disorders (2–5). Subsequently, the Hyperglycaemia and Adverse Pregnancy Outcome (HAPO) study reported a linear continuous relationship between maternal hyperglycaemia and perinatal adverse outcome, making it difficult to define clear diagnostic thresholds (6). Based on these results, the International Association of Diabetes and Pregnancy Study Groups (IADPSG) developed a new guideline in 2010 recommending a universal one-step diagnostic test using the OGTT 75 g between 24 and 28 weeks of gestation with only one value to be considered as abnormal (7). Using the new criteria, the prevalence of GDM increased in the HAPO study to approximately 18%, but varied widely among the different population demographics. For example, the prevalence in the HAPO study ranged between 9.3 and 25.5% dependent on the participating center (8). The new diagnostic thresholds have significant impact on costs and on infrastructure capacity. But many experts justify the criteria and the increase in workload in the background of the globally mounting burden of T2DM (9). The IADPSG thresholds were accepted by many health care organizations such as the WHO in 2013 and are now referred to as the 2013 WHO criteria (10). But the debate about screening and diagnostic criteria still goes on. The American Diabetes Association (ADA), which endorsed the IADPSG criteria in 2011, amended their guideline in 2014 and now considers both approaches (the one-step 75 g OGTT and the two-step screening: GCT followed by a 100 g OGTT if abnormal) acceptable for GDM diagnosis (11). The ADA states that there are insufficient data to demonstrate the superiority of one screening and diagnostic approach over the other, as—using the 2013 WHO criteria—the impact on costs and short and long term outcome of mother and her offspring have not been adequately evaluated. In their updated guideline from 2018, the American College of Obstetricians and Gynecologists (ACOG) recommends the two-step screening approach using the Carpenter and Coustan or the National Diabetes Data Group criteria and states however that “individual practices and institutions may choose to use the IADPSG recommendations” (12) The International Federation of Gynecology and Obstetrics (FIGO) acknowledged the problems with varying resource settings in different regions in their guideline from 2015 (13). The 2013 WHO criteria are generally recommended but the performance of OGTT vary depending on local circumstances. Diagnosis should be based on results from venous serum or plasma, but the use of plasma-calibrated handheld glucometers may be acceptable in locations where laboratory support is unavailable. While the 2013 WHO criteria are becoming more widely accepted, the main diabetes and obstetric societies still struggle to find the ideal algorithm. Large-scale randomized controlled trials studying the impact of intervention on women who meet different GDM criteria and evaluating the cost effectiveness of changes in short- and long-term outcomes might help solve these problems.

## Screening for overt diabetes in pregnancy and rationale for early GDM screening

Many health organizations recommend to test for overt diabetes in women at high risk at the first prenatal visit (7, 11). Women with overt diabetes in pregnancy suffer from a higher rate of vascular dysfunction. They have a higher risk of congenital malformation and a significantly increased risk of adverse pregnancy outcome (14). These women benefit the most from early treatment. However, early testing will also lead to the identification of hyperglycaemia under the threshold for overt diabetes in pregnancy. Like screening and diagnosis of GDM in late pregnancy, there is also no consensus on the diagnostic criteria for “early” GDM. Many experts do not recommend screening for GDM in the first trimester at all, as no valid data exists about the benefits and harms of diagnosing and treating GDM in early gestation (15). The aim of early testing would be mainly to identify women at low or high risk for GDM. This risk stratification would diminish the need for universal screening and diagnosis from 24 weeks onwards and would reduce workload and costs. The second goal would be to identify women who already have GDM and to start treatment as early as possible to adequately ameliorate the adverse short and long-term effects of prolonged intrauterine exposure to hyperglycaemia. Maternal hyperglycaemia occurring even before diagnosis of GDM after 24 weeks of gestation seems to already increase the rate of fetal growth (16) and—if treatment starts after 26 weeks—infant adiposity (17, 18). Recent studies suggest a long term risk for the offspring for T2DM and cardiovascular disease. GDM seems to influence DNA methylation involved in energy metabolism and anti-inflammatory processes. This “metabolic programming” might be modified by later intervention during pregnancy, but this needs to be elucidated in future studies. After assessment of GDM in first trimester—theoretically by now—early intervention would be of benefit for women who might be at highest risk for adverse pregnancy and long-term outcomes.

## Methods for early GDM screening

Many different methods have been evaluated for the screening of GDM in early pregnancy. There are direct glycaemic markers such as FPG, RPG, GCT, and/or OGTT, indirect methods like glycosylated hemoglobin A1c (HbA1c) or fructosamine and newer biochemical markers, many of which have been derived from proteomic or metabolomic analyses. We discuss the most promising approaches in detail below.

### Fasting and random plasma glucose in early pregnancy

Most health care organizations agree that screening for pre-conceptionally undiagnosed diabetes during pregnancy is recommendable, especially in high risk populations (19). Accordingly, the main advantage of FPG is its usefulness in diagnosing overt diabetes already at the first antenatal visit using standard diagnosis criteria [i.e., if FPG exceeds 125 mg/dl (6.9 mmol/l)] (7, 13, 20). Although the IADPSG consensus panel recommended in 2010 that GDM could be diagnosed by FPG concentrations between 92 and 125 mg/dl (5.1 and 6.9 mmol/l) at any time during gestation (including the first trimester), as well, this approach was criticized due to lack of evidence (20). First, the IADPSG thresholds considered diagnostic for GDM were derived from the HAPO study, where FPG and OGTT glucose levels were assessed in the late second and early third trimester (6). Moreover, one study from Asia showed a continuous fall of FPG values during first trimester (21). Hence, the IADPSG cut-offs are not necessarily applicable at earlier gestational periods (9). Second, there are no randomized clinical trials available supporting any benefit of treating GDM diagnosed before 24 weeks of gestation (19). Due to these concerns, some authors suggested that the use of the IADPSG threshold for FPG is not justified in early pregnancy and IADPSG representatives issued a statement in 2016 to discontinue use of the FPG threshold (15). However, higher first trimester FPG levels might be regarded as an independent risk factor for later GDM development, comparable to pre-gestational BMI (22). Indeed, previous studies indicated an association between first trimester FPG and GDM manifestation between 24 and 28 weeks of gestation by using the IADPSG cut-offs, with concordance measures (area under the receiver operating characteristic curve, ROC-AUC) ranging between 0.614 (23) and 0.654 (21), respectively. Of note, one recent retrospective study suggested that RPG assessed between 12 and 16 weeks is able to predict GDM according to various diagnostic criteria with an ROC-AUC of 0.80 (24). This seems to be surprisingly high compared to the concordance measures observed for FPG. Although results are conflicting (previous studies reported a less optimistic ROC-AUC of 0.69 for RPG assessed between 24 and 28 weeks of gestation) and RPG might be affected by several pre-test conditions such as food intake, it has several advantages regarding time and cost effectiveness (24, 25). Thus, future research including prospective confirmatory studies are necessary.

### OGTT in early pregnancy

An early OGTT (using an oral glucose load of 75 g glucose dissolved in 300 ml water) before 24 weeks of gestation could be also used for diagnosing overt diabetes if the 2-h plasma glucose level exceeds 199 mg/dl (11.1 mmol/l) according to FIGO and WHO guidelines (10, 13). Regarding diagnosis of GDM before 24 weeks of gestation the same concerns might be valid as discussed above for FPG. However, a recent study found that women meeting the IADPSG cut-offs already early in gestation showed impaired insulin sensitivity, which was partly explained by a higher degree of obesity in these patients (26). In accordance with these results, Lapolla et al. observed impaired insulin sensitivity in patients with early GDM diagnosis using the Carpenter-Coustan criteria (27).

### Glycosylated hemoglobin A1c in early pregnancy

HbA1c can be also used to detect overt diabetes at the first antenatal visit [≥6.5% (48 mmol/mol)] according to current guidelines. The test should be performed in a laboratory using a NGSP (National Glycohemoglobin Standardization Program) certified method standardized to the DCCT (Diabetes Control and Complication Trial) assay (20). Comparable to RPG, HbA1c has the advantage that it is inexpensive and does not require the fasting state. Fong and co-workers assessed its predictive performance for GDM progression in a retrospective cohort study which concluded that HbA1c levels between 5.7 and 6.4% (39–46 mmol/mol) could effectively identify patients at highest risk of developing GDM (28). A further study from New Zealand indicated that HbA1c ≥5.9% (41 mmol/mol) is highly predictive for pre-existing diabetes and adverse pregnancy outcomes (29). In addition, another study from Switzerland concluded that all pregnant women with first trimester HbA1c ≥6.0% (42 mmol/mol) developed GDM later in pregnancy, whereas those with HbA1c <4.5% (26 mmol/mol) did not (30). Conversely, Agarwal et al. found that the ROC-AUC of HbA1c assessed between 24 and 28 weeks was 0.54 and concluded that HbA1c remains a poor screening test for GDM using the WHO 1999 criteria (31). It might be of importance that HbA1c is subjected to pregnancy specific changes (32), requiring trimester specific reference values (33). Moreover, data from women after pregnancy with GDM indicated that HbA1c in the pre-diabetic range is a weak surrogate for the underlying pathophysiological components of impaired glucose metabolism, including impaired insulin action and β-cell dysfunction (34). Hence, HbA1c might be inferior to other tests for detecting subtle alterations in glucose metabolism.

### Other biochemical markers

**Figure 1:**
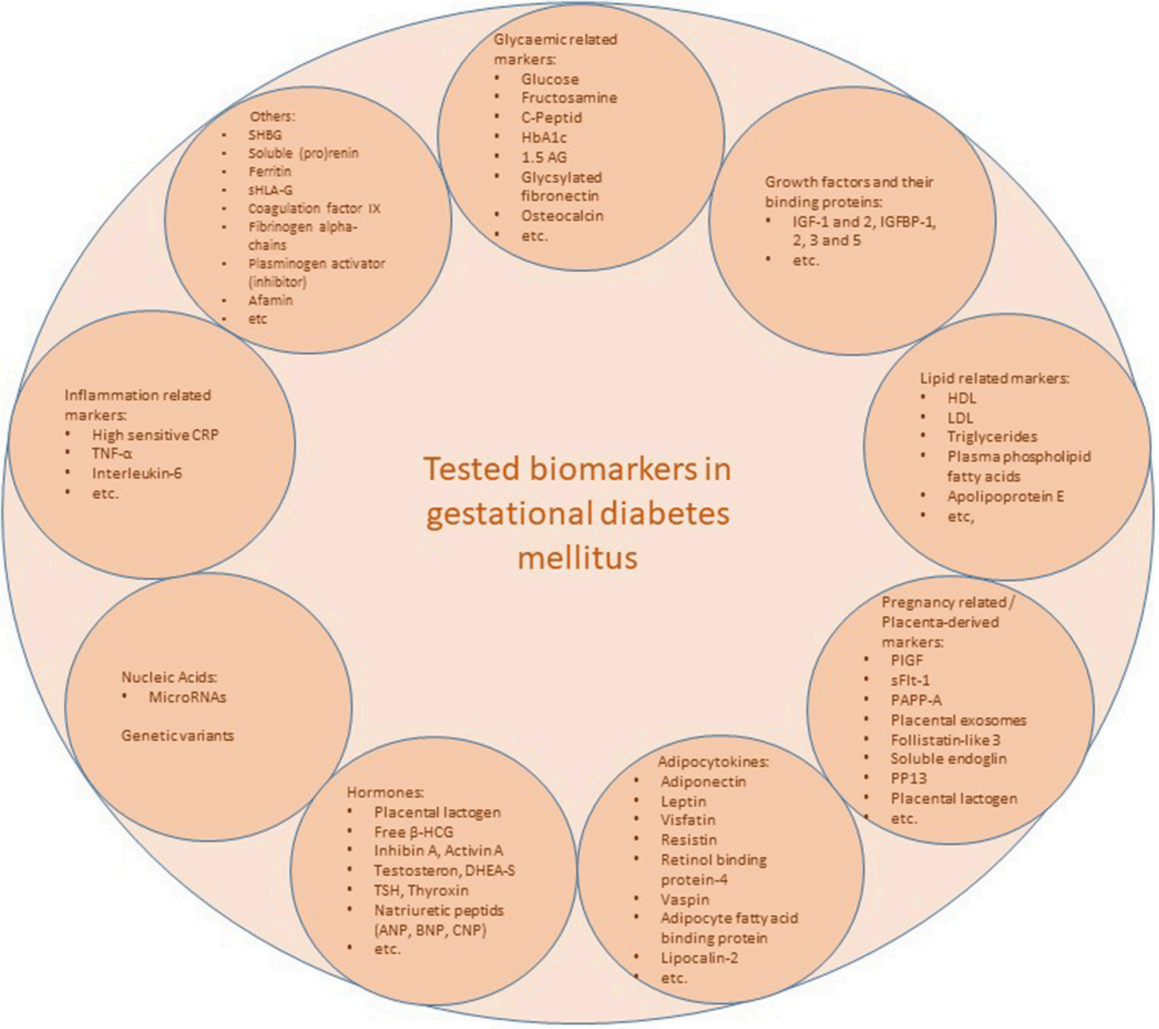
Overview of tested biomarkers. CRP, c-reactive protein; Free β-HCG, free β-human chorionic gonadotropin; GlyFn; glycosylated fibronectin; HbA1c, hemoglobin A1c; HDL, high density lipoprotein; hsCRP, high sensitive c-reactive protein; IGF, insulin like growth factor; IGFBP, insulin like growth factor binding protein; LDL, low density lipoprotein; MF, maternal factors; PAI-2, plasminogen activator inhibitor-2; PP13, placental protein 13; PAPP-A, pregnancy associated plasma protein-A; PlGF, Placental growth factor; microRNAs, micro ribonucleic acids; sFlt-1, soluble Fms-like tyrosine kinase-1; SHBG, sexual hormone binding globulin; sHLA-G, soluble human leucocyte antigen-G; TNF-α, tumor necrosis factor-α; TSH, thyroid-stimulating hormone; 1.5 AG, 1.5 Anhydroglucitol.

The current “gold standard” OGTT has a low reproducibility, is time consuming, unpleasant for some patients, dependent on ethnicity and the amount of glucose is given without consideration of maternal BMI (35). Therefore, the search for a simple, non-fasting point-of-care test or a risk model incorporating biomarkers (for a summary of risk models incorporating biomarkers ± maternal factors see Table 1) seems to be a logical consequence. Many biochemical markers in the first trimester have been evaluated (Figure 1), but often only in small case-control observations without further prospective validation. Some biomarkers such as fasting insulin, inflammatory markers such as C-reactive protein (CRP) or tumor necrosis factor-α (TNF-α) or soluble (pro)renin receptor failed to provide additional information about GDM risk beyond the risk assessment by clinical risk factors such as maternal BMI (47, 49, 50).

**Table 1:** Summary of biomarkers tested in a multivariate model for later development of gestational diabetes.

**Biomarker models**	**Gestational age (weeks)**	**Source**	**Population**		**Study design**	**Diagnostic criteria for GDM**	**Test performance measures**	**References**
			**GDM**	**No GDM**			
MF, PAPP-A, PlGF	11–13	Serum	787	30.438	Prospective cohort	WHO 1999	AUC 0.841, DR 58% at FPR 10%, (PAPPA and PlFG added no advantage)	Syngelaki et al. (36)
MF, PlGF, PAPP-A	11–14	Serum and Plasma	40	94	Case-control	WHO 2013	AUC 0.77, DR 34.3% at FPR of 10% (no difference of PAPP-A in both groups)	Eleftheriades et al. (37)
MF, PAPP-A, free β-HCG	11–13	Serum	248	732	Case-control	ADIPS 1998	AUC 0.90, DR 73.8% at FPR 10%	Sweeting et al. (38)
MF, triglycerides, lipocalin-2, PAPP-A, Leptin, Adiponectin, PAI-2	11–13	Serum	248	732	Case-control	ADIPS 1998	AUC 0.91, DR 76.8% at FPR 10% (Leptin, Adiponectin, and PAI-2 added no advantage)	Sweeting at al. (39)
MF, PAPP-A, adiponectin, PP13, endoglin	11–13	Serum	12	60	Case-control	WHO 2013	DR 63.6% at FPR of 10%, + BMI increased DR to 73% (no differences of PP13 or endoglin in both groups)	Farina et al. (40)
CRP, 1.5 AG, adiponectin, SHBG	6–15	Serum	46	178	Prospective high-risk cohort	WHO 2013	AUC of 1.5 AG 0.61; adiponectin (<8.9 ug/ml) OR (3.3 95%CI 1.65–9.67), SHBG had no link to GDM when corrected for BMI, ethnicity, or family history	Corcoran et al. (41)
MF, adiponectin, leptin	6–14	Serum	107	2483	Prospective cohort	OGTT 7 g 2 h >9 mmol/l	AUC 0.81 DR 44% at FPR of 10%	Thagaard et al. (42)
MF, adiponectin, SHBG, follistatin-like 3	11–13	Serum	80	300	Case-control	WHO 1999	AUC 0.84, DR 58.6% at FPR 10%, (no difference of follistatin like-3 in both groups)	Nanda et al. (43)
GlyFn, adiponectin, CRP, placental lactogen, SHBG	11–13	Serum	90	92	Case-control	WHO 1999	AUC 0.92, no association between SHBG and GDM, glyFn alone had a high AUC of 0.91 (Sens 81%, Spec 90%)	Rasanen et al. (44)
AG 1.5	13–23	Serum	50	50	Case-control	WHO 2013	AUC 0.951 (Sens 87%, Spec 94.1%)	Boritza et al. (45)
SHBG, hsCRP	6–15	Serum	27	242	Prospective observational study	Caprenter-Coustan	AUC 0.756, Sens 74.1% und Spec 75.6%	Maged et al. (46)
MF, hsCRP, TNF-α	11–13	Serum	200	800	Case-control	WHO 1999	AUC 0.82, DR 52% at FPR 10% using MF alone (hsCRP and TNF-alpha added no advantage)	Syngelaki et al. (47)
Apolipoprotein E, coagulation factor IX, fibrinogen alpha chain, IGFBP-5	12–16	Serum	30	30	Case-control	WHO 2013	AUC 0.985 (95%CI 0.958–1.012), sens 80% and spec 98%, Cave: Proteomic analysis, results have not validated yet in independent cohort!	Zhao et al. (48)

*AUC, area under the curve; BMI, body mass index; CRP, c-reactive protein; DR, detection rate; FPR, false positive rate; Free β-HCG, free β-human chorionic gonadotropin; GDM, gestational diabetes mellitus; GlyFn; glycosylated fibronectin; hsCRP, high sensitive c-reactive protein; IGFBP-5, insulin like growth factor binding protein-5; MF, maternal factors; OGTT, oral glucose tolerance test; PAI-2, plasminogen activator inhibitor-2; PP13, placental protein 13; PAPP-A, pregnancy associated plasma protein-A; PlGF, Placental growth factor; sens, sensitivity; SHBG, sexual hormone binding globulin; spec, specificity; TNF-α, tumor necrosis factor-α; WHO, world health organization; 1.5 AG, 1.5 Anhydroglucitol*.

The currently available marker, placenta growth factor (PlGF), is highly expressed by the placenta. Low PlGF levels in early pregnancy as a sign of poor placentation were observed in women with preeclampsia and/or intrauterine growth restriction (IUGR) and PlGF is now widely used for predicting preeclampsia at the time of the aneuploidy screening in 11–14 weeks of gestation (51). In a small case-control study, Eleftheriades et al. could show an increase of PlGF in early pregnancy in women with GDM compared to unaffected pregnant women (37). A risk prediction model with maternal factors alone could be improved from an AUC of 0.73 to 0.77 by the addition of PlGF but not pregnancy associated plasma protein-A (PAPP-A). On the other hand, a large prospective cohort study from the UK with over 31,000 recruited women showed only little advantage in incorporating PlGF and PAPP-A to a risk model which included maternal factors (AUC 0.84) (36). Conflicting results also exist for PAPP-A. Some studies report that PAPP-A levels are decreased in early pregnancy in women who subsequently developed GDM (52–55), others show no differences in comparison to normal women (56, 57). Sweeting et al. reported a lower PAPP-A level especially in women of South Asian ethnicity and in multiparous women (38). The addition of aneuploidy markers increased the predictive value with an AUC of 0.88% by maternal factors alone to 0.90% with a detection rate (DR) of 73.8% at a false positive rate (FPR) of 10%.

Adipocytokines such as adiponectin and leptin are hormones secreted by adipose tissue. Adiponectin plays an important role in glucose regulation and seems to be a good marker for whole body insulin sensitivity (58). A recent meta-analysis incorporating eight studies using early pregnancy adiponectin levels suggested moderate predictive ability of adiponectin in the prediction of GDM with an AUC of 0.79, a sensitivity of 60.3% (95% CI 46,0%, 73.1%) and a specificity of 81.3% (95% CI 71.6%, 88.3%) (59). Leptin regulates energy intake and expenditure and its levels correlate with the amount of visceral fat in first trimester (60). Qiu et al. found that each 10 ng/ml increase in leptin level was associated with a 20% higher risk of GDM (61), but others reported no alterations in leptin levels in women who subsequently developed GDM (62) or only an association in severely obese women (42). The latter study reported an AUC of 0.82 with a DR of 42% at a FPR of 10% in normal weight and moderately obese women when adiponectin and leptin were included in the maternal factor-based risk model. In a moderate sized case-control study (44), first trimester adiponectin and a newly introduced biomarker glycosylated fibronectin (GlyFn) were independently associated with later GDM development after adjustment for maternal clinical parameters (but not maternal BMI) with an AUC of 0.91 for GlyFn and an AUC of 0.63 for adiponectin. The glycated protein GlyFn still needs to be further validated in large prospective studies. Another marker of short-term glycaemic control is 1.5 Anhydroglucitol (1.5 AG). 1.5 AG is the 1-deoxy form of glucose and is a marker for short term (prior 24–72 h) glycaemic control and variances. Boritza et al. (45) could show that 1.5 AG could discriminate women with GDM and normal women before 20 weeks of gestation in a small case-control study. 1.5 AG had a high predictive value with an AUC of 0.951, a sensitivity of 87% and a specificity of 94.1% with a cut-off of ≤ 60.3 umol/l.

Nonetheless, new biomarkers will need several years to evaluate and large-scale prospective observational studies to prove their clinical utility, as well as, to assess their cost effectiveness in comparison to later GDM screening and diagnosis are necessary. Additionally, it is unlikely that only one biomarker will have high enough sensitivity and specificity to assess early maternal hyperglycaemia. It is more likely that the combination of multiple parameters including baseline maternal characteristics such as BMI will achieve adequate predictive performances, as is the case with first trimester screening for aneuploidy or more recently for preeclampsia. Additionally, the fast-developing field of “omics,” in particular proteomic and metabolomic analyses, will provide deeper insights into the pathophysiology of the different phenotypes of gestational diabetes. A specific protein or metabolite pattern will help to decipher biological processes in a more holistic way. These metabolic “fingerprints” of different body fluid sources could then be used for predictive purposes as has already been demonstrated in small case-control studies (48, 63).

### Risk factor-based screening

Clinical risk factors for GDM such as higher maternal age, obesity, GDM in a previous pregnancy, family history of diabetes, glycosuria, and ethnic background could be used in combination to identify women with increased risk of developing GDM. However, the proposed clinical risk indicators have shown limited diagnostic accuracy when used separately (64, 65). Therefore, some authors suggested that sensitivity and specificity for GDM screening with risk factors could be considerably improved by using clinical risk prediction models that include statistical combinations of several risk indicators (43, 65–70), which might be additionally combined with FPG (71, 72). Moreover, other biochemical markers might be included for improved prediction (73). However, the design of sufficient risk scores requires an adequate number of cases and healthy controls. As another limitation, the association of different risk factors (e.g., BMI) varies between different ethnic groups (74). Moreover, external validation in clinical practice is necessary (but often pending). A large number of risk estimation models for GDM can be found in the current literature, whereby most of them are based on different diagnostic criteria (65–68, 71). While they might be applicable even in early gestation to identify women at particularly high risk, their clinical significance has not been examined, or compared in independent populations. Furthermore, there is strong evidence from large epidemiological studies (75) that adherence to a healthy life-style (physical activity, healthy diet, non-smoker) prior to gestation is strongly associated with a lower risk for GDM. However, data on life-style factors is missing in published risk scoring algorithms, indicating the need for further research on this topic.

## Maternal characteristics of early vs. late GDM

An overlap between the categorization of GDM and overt diabetes can be present if overt diabetes had not already been diagnosed before pregnancy. This in mind, pregnant women diagnosed with GDM in early pregnancy seem to be associated with worse pregnancy outcomes approximating those seen in overt diabetes (18). The data from Sweeting et al. suggests a heterogeneous “early” GDM phenotype with a continuous risk from overt diabetes in pregnancy to early GDM, with GDM diagnosed from 24 weeks of gestation onwards being the lowest risk condition. Additionally, maternal adiposity and a more insulin-resistant phenotype might also play a role in the heterogeneity of “early” GDM (26).

## Conclusion and recommendations

GDM is currently diagnosed during the second or third trimester, when unfavorable metabolic dysfunctions might have already affected the mother and the fetus. The combination of maternal risk factors and the insulin resistance preceding biomarkers might improve early detection of a high risk GDM cohort. Future studies should evaluate whether early GDM screening and diagnosis can be improved with the addition of novel biomarkers implicated in the pathophysiology of GDM, whether earlier detection and intervention strategies can improve short and long term adverse outcome and whether the combined biomarker and maternal factor screening models are cost-effective.

## Author contributions

EH and CG drafted manuscript. SR and IH made substantial contribution to the content.

### Conflict of interest statement

The authors declare that the research was conducted in the absence of any commercial or financial relationships that could be construed as a potential conflict of interest.
